# Characterization of Fatty Acid, Amino Acid and Volatile Compound Compositions and Bioactive Components of Seven Coffee (*Coffea robusta*) Cultivars Grown in Hainan Province, China

**DOI:** 10.3390/molecules200916687

**Published:** 2015-09-14

**Authors:** Wenjiang Dong, Lehe Tan, Jianping Zhao, Rongsuo Hu, Minquan Lu

**Affiliations:** 1 Spice and Beverage Research Institute, Chinese Academy of Tropical Agricultural Sciences (CATAS), Wanning 571533, Hainan, China; E-Mails: dongwenjiang.123@163.com (W.D.); tlh3687@163.com (L.T.); hnhrs@126.com (R.H.); lmq663@126.com (M.L.); 2 National Center of Important Tropical Crops Engineering and Technology Research, Wanning 571533, Hainan, China

**Keywords:** Robusta coffee, cultivar differentiation, chemical composition, chemometric techniques, HS-SPME/GC-MS

## Abstract

Compositions of fatty acid, amino acids, and volatile compound were investigated in green coffee beans of seven cultivars of *Coffea* *robusta* grown in Hainan Province, China. The chlorogenic acids, trigonelline, caffeine, total lipid, and total protein contents as well as color parameters were measured. Chemometric techniques, principal component analysis (PCA), hierarchical cluster analysis (HCA), and analysis of one-way variance (ANOVA) were performed on the complete data set to reveal chemical differences among all cultivars and identify markers characteristic of a particular botanical origin of the coffee. The major fatty acids of coffee were linoleic acid, palmitic acid, oleic acid, and arachic acid. Leucine (0.84 g/100 g DW), lysine (0.63 g/100 g DW), and arginine (0.61 g/100 g DW) were the predominant essential amino acids (EAAs) in the coffee samples. Seventy-nine volatile compounds were identified and semi-quantified by HS-SPME/GC-MS. PCA of the complete data matrix demonstrated that there were significant differences among all cultivars, HCA supported the results of PCA and achieved a satisfactory classification performance.

## 1. Introduction

Coffee is an evergreen arbor of the Rubiaceae family as well as one of the most consumed beverages in the world [1,2], and green coffee beans are now produced in more than 70 countries [3]. The most cultivated varieties are *Coffea arabica* (*Arabica*) and *Coffea canephora* (*Robusta*), which are used for commercial production, accounting for about 60% and 40% of the world coffee market, respectively, while *Coffea liberica* Bull ex Hiern contributes less than 1% of marketed coffee [4,5,6]. The quality of each coffee species varies with its cultivars, geographical origin and climatic environment such as altitude, temperature, fertilization and soil. Recently, with the growing recognition on the importance of green coffee beans to potential health benefits for consumers, its unique composition and properties have received great attention [7].

Hainan Province of China, is a well-known cultivation base of *Coffea canephora* [8]. Xinglong and Fushan coffees are the most popular coffees of China, protected by the Chinese AQSIQ (the People’s Republic of the China’s FDA) “Controlled Designation of Origin” [9]. There has been a constant increase in consumption since it was first cultivated in the 1960s, and the cultivation area is mainly distributed in the Wanning, Chenmai, Sanya, and Baisha municipal regions. In recent years, Chinese farmers have cultivated new coffee cultivars with excellent adaptability to the local geographical environment conditions. However, different cultivars show variations in chemical composition of the coffee fruits, leading to flavor quality differences in the corresponding roasted coffee beans [10,11].

Green coffee beans (GCB) are rich in bioactive compounds, especially caffeine, trigonelline, and chlorogenic acids (CGAs) [12], and a number of functional properties of green coffee beans has been reported. Caffeine presents various biological activities such as stimulation of the central nervous system, myocardial stimulation, and peripheral vasoconstriction [13]. Scientific studies have suggested that trigonelline was capable of stimulating respiratory activity and promoting glucose utilization in HepG2 cells [14]. Chlorogenic acids, the main phenolics in green coffee, are known to have anti-inflammatory, antibacterial, and anticancer activities and DNA protective functions, *etc*. [15,16,17]. Polyphenolic and hydroxycinnamate contents have been identified and quantified in the whole coffee fruits from China, India, and Mexico by Mullen *et al.* [18] Smarke *et al.* [19] reported that chlorogenic acids and coffee may help in preventing retinal degeneration. However, no studies can be found for the differences of contents, caffeine, trigonelline, and chlorogenic acids, among different Robusta coffee cultivars. The fat and protein, which are flavor precursors of roasted coffee, are important components of green coffee beans, and the lipid fraction of green coffee beans comprises mainly of triacylglycerols, sterols, tocopherols, and diterpenes of the kaurene family [20,21]. Fatty acids are beneficial to human health since they are precursors in the biosynthesis of eicosanoids, which are proposed as bioregulators of many cellular metabolic processes [22,23]. It was found that green coffee beans are rich in unsaturated fatty acids such as oleic (18:1n-9), linoleic (18:2n-6), and linolenic (18:3n-3) acids. Triacylglycerols (TAG) represent the main lipid class in coffee samples. Fatty acid composition was also applied to differentiate between Arabica and Robusta coffee in a mixture [24,25]. Proteins are composed of different amino acids which are the main compounds contributing to the formation of the typical aroma during roasting, and previous studies have reported that alanine is the amino acid with the highest content (1.2 mg/g) in Robusta coffee beans, followed by asparagine (0.68 mg/g) [26]. Casal *et al.* [27] previously verified that d- and l-amino acids have the potential to be used as coffee species discriminators. The fatty acid and amino acids profiles of coffee beans from some regions of the world have been well documented in the literature. Nevertheless, to our knowledge, studies about the fatty and amino acids composition of Chinese Robusta coffee are not available. Generally, the volatile composition may vary according to genetics, soil, climate, and agricultural practices. Bertrand *et al.* [28] investigated the influence of climatic conditions on the volatile compound fingerprint in green Arabica coffee beans and coffee beverage quality; Toci *et al.* [29] analyzed the volatile fingerprint of defective Brazilian coffee seeds to corroborate potential marker compounds and identify new low quality indicators. The previous studies mainly focused on the agronomic characteristic and cultivation technique, few were dedicated to chemical composition of green coffee beans according to different cultivars.

The aim of this study was to analyze and evaluate the quantitative and bioactive characteristics of seven different Chinese Robusta coffee cultivars in terms of the color, chlorogenic acids, trigonelline, caffeine, protein, fat, fatty acid, and amino acid composition, as well as the volatile organic compounds of coffee samples. Results from this study will provide new knowledge about the bioactive ingredient and flavor precursor composition of Robusta coffee, and will be helpful to agronomists in their search for the best-adapted and most optimal coffee cultivars.

## 2. Results and Discussion

### 2.1. Color Characteristics

Color is often the first characteristic noted in a food, and usually affects the consumer’s attitudes towards food flavor and quality. The color was calculated by tristimulus colorimetry, and the values of L*, a*, b*, C*, H° and $∆{\text{E}}_{\text{ab}}^{\text{*}}$ of the seven cultivars presented in Table 1 indicated that there were significant differences among the cultivars. The L* values indicate the lightness of color (positive = 100, negative = 0), it can be observed that “RY1” presented the darkest value of 59.31 ± 0.72, and X1 had the lightest value of 53.35 ± 0.36. Variation in L* value was also previously reported by Craig *et al.* [30] With respect to a* value, which indicates how color varies from redness (positive) to greenness, while the highest content of red color corresponded to “X24-2” and the lowest to “X1”, significant differences in a* value were found among all cultivars except for “RY1”, “X26”, and “RY2”. The b* value indicates the yellowness (positive b*) and blueness (negative b*), and “RY2” showed the highest value of 25.36 ± 0.13 while the lowest value of 20.90 ± 0.05 was seen for “XCM”. Chroma (C*) and hue angle (H°) could provide more information about the spatial distribution of colours than direct values of tristimulus measurements, and results for C* were similar to the b* values for ground coffee samples, whereas “X1” had the highest and “X24-2” the lowest value for H°. Since total colour difference ($∆{\text{E}}_{\text{ab}}^{\text{*}}$) is a function of the three L*, a*, and b*, changes from 5.26 ± 0.48 to 0.97 ± 0.29 were calculated for the “RY1” and “X1” samples, respectively.

**Table 1:** Color parameters of seven Robusta coffee cultivars.

Cultivar	L*	a*	b*	C*	H*	$∆E_{ab}^{*}$
**X1**	53.35 ± 0.36 ^e^	3.23 ± 0.06 ^f^	24.77 ± 0.97 ^ab^	24.98 ± 0.97 ^ab^	83.05 ± 0.07 ^a^	0.97 ± 0.29 ^d^
**RY1**	59.31 ± 0.72 ^a^	4.82 ± 0.23 ^b^	22.78 ± 0.30 ^d^	23.29 ± 0.29 ^d^	81.15 ± 0.24 ^d^	5.26 ± 0.48 ^a^
**RY2**	56.57 ± 0.32 ^c^	4.55 ± 0.13 ^c^	25.36 ± 0.13 ^a^	25.77 ± 0.10 ^a^	81.82 ± 0.15 ^bc^	3.31 ± 0.22 ^c^
**X24-2**	54.32 ± 0.19 ^de^	5.52 ± 0.08 ^a^	23.65 ± 0.19 ^cd^	24.30 ± 0.21 ^bc^	80.58 ± 0.06 ^e^	2.98 ± 0.06 ^c^
**X26**	58.21 ± 1.05 ^b^	4.60 ± 0.11 ^bc^	24.12 ± 0.68 ^bc^	24.56 ± 0.68 ^bc^	81.59 ± 0.12 ^c^	4.89 ± 0.69 ^b^
**X28**	55.36 ± 0.48 ^d^	4.22 ± 0.13 ^d^	23.34 ± 0.44 ^cd^	23.72 ± 0.46 ^cd^	81.86 ± 0.07 ^b^	2.63 ± 0.40 ^c^
**XCM**	54.54 ± 0.73 ^d^	3.70 ± 0.19 ^e^	20.90 ± 0.05 ^e^	21.23 ± 0.07 ^e^	82.02 ± 0.20 ^b^	4.20 ± 0.14 ^b^

Means with different superscript letters (a, b, c, d, e) within same row are significant different (*p* < 0.05).

### 2.2. Chlorogenic Acids, Trigonelline, and Caffeine Analysis

**Figure 1:**
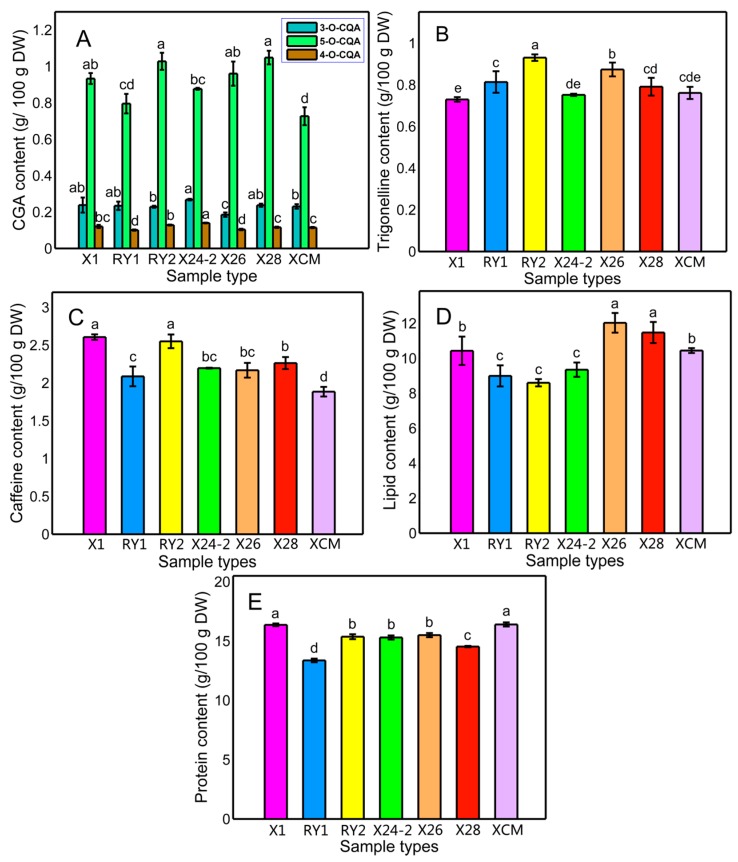
Chlorogenic acids (**A**), trigonelline (**B**); caffeine (**C**); lipid (**D**); and protein (**E**) contents of seven cultivars of Robusta coffee. Vertical bars represent standard deviations. Different letters indicate significant differences at *p* < 0.05.

Chlorogenic acids are important chemical components in green coffee beans, the most common being caffeic acid, ferulic acid, and *p*-cumaric acid which form esters with quinic acid, as has been widely reported in the literature [18]. Quantification of chlorogenic acids, trigonelline, and caffeine was performed by comparison with their respective standards. Figure 1A shows the chlorogenic acids (3-*O*-CQA, 5-*O*-CQA, and 4-*O*-CQA) for each cultivar of green coffee beans. No significant differences were found in the content of 3-*O*-CQA between the seven cultivars. 5-*O*-CQA was the major CGA in all samples, and the “X28” and “RY2” cultivars showed the highest content of 5-*O*-CQA (from 1.05 to 1.03 g/100 g DW), followed by the “X26” and “X1” cultivars (from 0.96 to 0.93 g/100 g DW), and the “X24-2”, “RY1”, and “XCM” cultivars had the lowest content of 5-CQA, ranging from 0.73 to 0.88 g/100 g DW. With the respect to the content of 4-*O*-CQA, the highest was found in “X24-2” (0.14 g/100 g DW), while “X26” presented the lowest (0.10 g/100 g DW). There were statistically significant differences between the “X24-2”, “RY2”, “X28”, and “RY1” cultivars. Other authors have also reported the content of CGA in green coffee beans from different cultivars, finding that the content of 3-*O*-CQA, 5-*O*-CQA, and 4-*O*-CQA ranged from 0.2 to 1.3 mg/g DW, 1.3 to 11.0 mg/g DW, and 0.4 to 2.5 mg/g DW for cultivars of *C. canephora*, respectively. Those results were in agreement with the reported work [18]. On the other hand, Smrke *et al.* [31] reported contents of 0.17%–0.21%, 2.9%–3.2%, and 0.28%–0.34% of 3-*O*-CQA, 5-*O*-CQA, and 4-*O*-CQA, respectively, estimated from HPLC-DAD results based on ethanolic extracts, and those values coincide with the results in this study. Differences between the contents of CGA could be attributed to the geographical factors of samples such as soil, elevation, climate, *etc*. and agricultural practices. The quantitative results of trigonelline are presented in Figure 1B. The ranges observed in this study were from 0.75 to 0.87 g/100 g DW. Several papers have investigated the content of trigonelline of coffee from around the world and our results were similar to the literature reports [32,33]. The content of caffeine in the green coffee beans from seven cultivars is shown in Figure 1C, where it can be observed that significant differences were found between most cultivars, as samples from the “X1” and “RY2” cultivars had similarly high quantities of caffeine (2.61 and 2.55 g/100 g DW, respectively), followed by the “X28”, “X24-2”, and “X26” cultivars (2.26, 2.20, and 2.17 g/100 g DW, respectively), whereas the samples from ‘RY1” and ‘XCM” were much lower (2.08 and 1.88 g/100 g DW, respectively).

### 2.3. Total Lipid Content

The total lipid content of different coffee cultivars ranged from 8.60 to 12.03 g/100 g DW (Figure 1D). The highest content was found in “X26” cultivar and the lowest was “RY2”, with the presence of three statistically different clusters of samples: “X26” and “X28” showed the higher content of lipids 12.03 and 11.48 g/100 g DW, respectively, whereas “XCM” and “X1” cultivars displayed an intermediate content of total lipids (10.44 and 10.41 g/100 g DW, respectively), and the “X24-2”, “RY1”, and “RY2” cultivars showed the lowest contents of lipids (9.35, 8.90, and 8.60 g/100 g DW, respectively). No significant difference in lipid content was found among the “X24-2”, “RY1”, and “RY2” samples (*p* < 0.05). Obviously, the lipid content was influenced by coffee cultivars. Previous studies have reported that total lipid content was 13.0–17.0 g/100 g DW in green coffee beans [34,35], which was higher than that of our report. Oliveira *et al.* [36] determined the amounts of lipid extracted from defective beans and healthy mature beans, and the lipid contents were found to be significantly different among health and defective beans according to the Duncan test at 5% probability, with the lipid content ranging from 9.2 to 10.8 g/100 g DW. Our results were consistent with those results. In an early report, Ramalakshmi *et al.* [37] found that the lipid contents of defective coffee beans (7.2% to 12.7%) were lower than those of the corresponding category of graded coffee beans (8.7% to 16.3%).

### 2.4. Total Protein Content

Proteins are known to important flavor precursors in the coffee bean roasting process. During roasting, proteins are denatured and fragmented [38]. Interchange reactions between disulfide aroma compounds and the protein sulfhydryl groups have also been reported [39]. The protein content of the seven coffee cultivars analyzed is presented in Figure 1E, where significant differences can be observed among cultivars at *p* < 0.05. It can be noticed that the “XCM” cultivar contained the highest crude protein (16.4 g/100 g DW), and the ‘RY1” cultivar contained the least of the seven cultivars studied (13.4 g/100 g DW). No significant difference was found between the “X26”, “RY2”, and “X24-2” cultivars, which varied from 15.3 to 15.5 g/100 g DW, which corresponds to the intermediate level among all cultivars. Our results were in consistent with a previous study of several commercial varieties of coffee cultivated in Brazil (14.9–17.0 g/100 g DW) [36]. Vasconcelos, *et al.* [40] compared the protein content between defective green and roasted coffee beans, and they found that protein levels for green coffee beans were in the range of 8.1 to 10.2, and no significant differences were detected between defective and non-defective beans. Our protein levels were slightly higher than that of literature reports, a phenomenon attributed to the different geographical origins of the coffee samples.

### 2.5. Fatty Acid Composition

**Figure 2:**
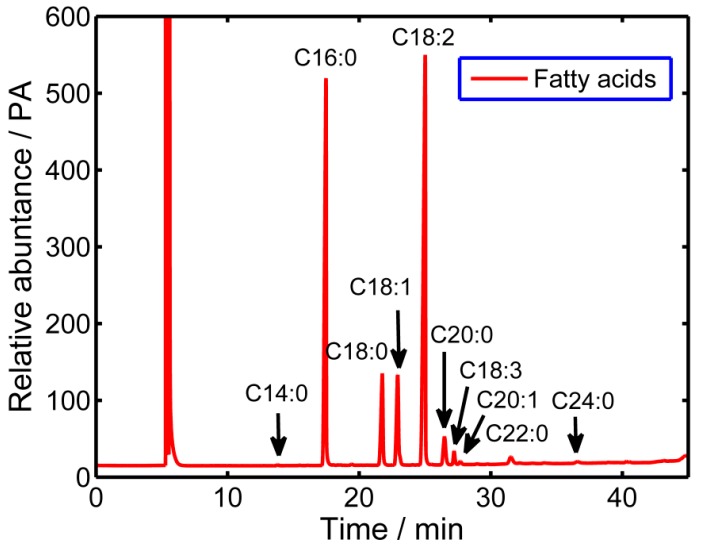
Representative GC-MS chromatogram for the analysis of fatty acids in Robusta coffee.

The fatty acid composition of the seven coffee cultivars is given in Table 2. The major fatty acids in the coffee oil analyzed in this study were linoleic acid (C18:2), palmitic acid (C16:0), oleic acid (C18:1), and arachic acid (C20:0), followed by small amounts of linolenic acid (C18:3), docosanoic acid (C22:0), tetracosanoic acid (C24:0), eicosenoic acid (C20:1), myristic acid (C14:0), and tricosanoic acid (C23:0). All fatty acids above were detected in the seven coffee cultivars. A characteristic fatty acid chromatogram is presented in Figure 2. In general, our results were similar to previous studies that proved C18:2 and C16:0 were the predominant fatty acids in green coffee oil. In all of them, more than 54% of total fatty acids (TFA) of the coffee bean were unsaturated, the remaining were saturated (46%). Total unsaturated fatty acids (ΣUFA) ranged between 1122.7 mg/100 g DW (“RY1”) and 2040.2 mg/100 g DW (“X26”), total saturated fatty acids (ΣSFA) ranged between 1076.8 mg/100 g DW (“RY1”) and 1766.4 mg/100 g DW (“X26”), while TFA had a value from 2199.5 mg/100 g DW (“RY1”) to 3806.6 mg/100 g DW (“X26”). The UFA linoleic acid was the most abundant fatty acid, and noticeable differences in linoleic acid content were observed, with the greatest content determined in “RY1” (920.0 mg/100 g DW) and the lowest content in “X26” (1600.0 mg/100 g DW). Linoleic acid is an essential fatty acid required by humans from external sources, which could contribute to decrease the ratio of low-density lipoproteins to high-density lipoproteins [41]. The SFA linolenic acid was the second most abundant fatty acid, with a value from 780.0 to 1300.0 mg/100 g DW. The ratios of ΣUFA/ΣSFA were larger than 1.0 for all cultivars, especially in “XCM” (1.40 mg/100 g DW), which indicated that green coffee beans could serve as a food supplement in the diet to decrease the level of fats and cholesterol, preventing some cardiovascular diseases [42]. Results were in accordance with previous reports than coffee beans contained much more unsaturated fatty acids [25]. Martin *et al.* [43] suggested that the amounts of myristic, oleic, linoleic and linolenic acids could be used as possible discriminating factor between Arabica and Canephora varieties. Accordingly, the four fatty acids above were the major fatty acids of the seven Robusta coffee beans cultivars studied in our paper.

**Table 2:** Fatty acid composition (mg/100 g DW) of Robusta coffee cultivars grown in China.

Fatty Acids	Cultivars						
	X1	RY1	RY2	X24-2	X26	X28	XCM
**Saturated Fatty Acids (SFAs)**							
**C14:0**	5.6 ± 0.3 ^c^	10.7 ± 1.5 ^b^	9.5 ± 0.2 ^b^	14.9 ± 2.0 ^a^	9.7 ± 0.1 ^b^	10.5 ± 0.9 ^b^	10.6 ± 0.8 ^b^
**C16:0**	1200.0 ± 13.0 ^b^	780.0 ± 10.0 ^e^	833.3 ± 25.2 ^de^	853.2 ± 70.9 ^d^	1300.0 ± 12.1 ^a^	953.4 ± 32.1 ^c^	913.3 ± 15.3 ^c^
**C18:0**	260.0 ± 4.0 ^b^	150.0 ± 3.2 ^f^	163.3 ± 5.8 ^e^	186.7 ± 5.8 ^d^	273.4 ± 5.7 ^a^	196.5 ± 11.5 ^d^	213.3 ± 5.8 ^c^
**C20:0**	150.0 ± 1.0 ^a^	95.4 ± 0.5 ^d^	98.0 ± 3.0 ^cd^	101.7 ± 7.6 ^cd^	130.0 ± 1.0 ^b^	100.0 ± 1.7 ^cd^	103.3 ± 5.8 ^c^
**C22:0**	28.1 ± 1.1 ^a^	20.3 ± 0.6 ^c^	24.2 ± 0.6 ^b^	24.3 ± 2.5 ^b^	29.6 ± 0.5 ^a^	24.0 ± 1.1 ^b^	23.4 ± 0.7 ^b^
**C23:0**	5.5 ± 0.5 ^c^	5.8 ± 0.2 ^c^	7.3 ± 0.2 ^b^	8.5 ± 1.0 ^a^	8.1 ± 0.2 ^ab^	6.4 ± 0.4 ^c^	8.7 ± 0.3 ^a^
**C24:0**	18.0 ± 4.4 ^ab^	14.6 ± 2.8 ^bc^	19.3 ± 0.6 ^a^	12.4 ± 0.6 ^cd^	15.6 ± 1.2 ^abc^	13.3 ± 0.5 ^cd^	9.8 ± 0.3 ^d^
**Unsaturated Fatty Acids (UFAs)**							
**C18:1**	376.6 ± 5.7 ^a^	170.0 ± 1.0 ^f^	226.4 ± 5.7 ^e^	260.0 ± 20.1 ^d^	366.5 ± 11.5 ^a^	286.8 ± 15.3 ^c^	313.4 ± 15.2 ^b^
**C18:2**	1600.0 ± 27.2 ^a^	920.0 ± 20.0 ^e^	1070.0 ± 60.8 ^d^	1013.2 ± 80.8 ^de^	1633.3 ± 57.7 ^a^	1266.5 ± 57.6 ^c^	1433.2 ± 57.5 ^b^
**C18:3**	26.5 ± 0.6 ^b^	24.6 ± 1.5 ^c^	20.2 ± 0.6 ^d^	17.0 ± 1.0 ^e^	27.7 ± 0.5 ^b^	23.5 ± 1.1 ^c^	35.4 ± 0.6 ^a^
**C20:1**	14.3 ± 0.6 ^a^	8.1 ± 0.1 ^e^	8.7 ± 0.4 ^de^	9.2 ± 0.3 ^d^	12.7 ± 0.6 ^b^	10.6 ± 0.5 ^c^	13.2 ± 0.6 ^b^
**ΣSFA**	1667.2 ± 24.3	1076.8 ± 18.8	1154.9 ± 35.6	1201.7 ± 90.4	1766.4 ± 20.8	1304.1 ± 48.2	1282.4 ± 29.0
**ΣUFA**	2017.4 ± 34.1	1122.7 ± 22.6	1325.3 ± 67.5	1299.4 ± 102.2	2040.2 ± 70.3	1587.4 ± 74.5	1795.2 ± 73.9
**TFA**	3684.6 ± 58.4	2199.5 ± 41.4	2480.2 ± 103.1	2501.1 ± 192.6	3806.6 ± 91.1	2891.5 ± 122.7	3077.6 ±102.9
**ΣUFA/ΣSFA**	1.21 ± 0.01	1.04 ± 0.01	1.15 ± 0.03	1.08 ± 0.02	1.15 ± 0.02	1.22 ± 0.04	1.40 ± 0.02

Fatty acids content are expressed as mg/100 g DW, means with different letters within same row are significant different (*p* < 0.05). ΣSFA = total saturated fatty acids; ΣUFA = total unsaturated fatty acids; TFA = total fatty acids.

### 2.6. Amino Acid Composition

Amino acid compositions of all the samples are reported in Table 3. To reveal cultivar-specific differences between different samples, statistical analysis (ANOVA, *p* < 0.05) of all amino acids data was subsequently performed based on the mean values of all cultivars. Essential amino acids (EAAs) accounted for 3.28%‒4.08% of the total, and the rest were non-essential amino acids (NEAs). Significant differences were found among the coffee cultivars. Leucine (0.74 to 0.96 g/100 g DW, mean value 0.84 g/100 g DW), lysine (0.58 to 0.69 g/100 g DW, mean value of 0.63 g/100 g DW), and arginine (0.54 to 0.66 g/100 g DW, mean value of 0.61 g/100 g DW) were the predominant EAAs in coffee samples. Phenylalanine, isoleucine and threonine were present in small amounts at 0.50%‒0.67%, 0.34%‒0.44%, and 0.32%‒0.39%, respectively. Methionine and histidine were in trace amounts of 0.05%‒0.07% and 0.19%‒0.21%, respectively. Glutamic acid and aspartic acid at 1.84%‒2.40% and 0.94%‒1.06%, respectively, turned out to be the predominant NEAs. Glycine, valine, proline and serine were present in small amounts of 0.58%‒0.70%, 0.49%‒0.62%, 0.46%‒0.59% and 0.45%‒0.55%, respectively, whereas tyrosine and alanine were in trace amount at 0.22%‒0.27% and 0.43%‒0.57%. The EAA contents indicated that the “RY1” sample possessed the lowest percentage (3.28%) of TAAs, followed by “X28” (3.53%), “X24-2” (3.59%), “X26” (3.65%), “RY2” (3.68%), and “X1” (3.75%), while the highest content was found in “XCM” (4.08%). The contents of NEAs varied from 5.42% (“RY1”) to 6.85% (“XCM”). The ratios of NEAs to total amino acids (TAA) ranged from 0.59% in “XCM” to 0.62% in the ‘X26” cultivar, and the TAAs varied from 8.70% to 10.59%. The results of this study were in accordance with a previous study, which reported that the TAAs of Robusta coffee were 7.025 g/100 g DW, and our results was higher than those reported by Casal *et al.* [27]. In addition, the amino acid composition were somewhat different from that in Indian coffee [26], where alanine had the highest content, from 0.41 to 1.40 g/100 g DW, followed by asparagine ranging from 0.28 to 0.96 g/100 g DW, and phenylalanine varied from 0.18 to 0.78 g/100 g DW. Casal *et al.* [44] investigated the effect of roasting on the free and bound amino acids (both d- and l- enantiomers) to compare the susceptibilities of Arabica and Robusta coffee to amino acid racemization, The results showed that an increase in the racemization value with temperature was observed, with aspartic acid being the most sensitive amino acid. In our samples, glutamic acid, aspartic acid, leucine acid, and lysine were the predominant amino acids. Perhaps the different growing environments or the varieties and storage conditions result in differences in the amino acid compositions of coffee samples.

**Table 3:** Amino acid composition of seven cultivars of Robusta coffee (g/100 g DW, *n* = 3).

Amino Acid	X1	RY1	RY2	X24-2	X26	X28	XCM
**Essential Amino Acids (EAA)**							
**Arginine**	0.62 ± 0.05 ^ab^	0.54 ± 0.05 ^b^	0.62 ± 0.05 ^ab^	0.61 ± 0.08 ^ab^	0.61 ± 0.04 ^ab^	0.58 ± 0.04 ^ab^	0.66 ± 0.05 ^a^
**Leucine**	0.85 ± 0.03 ^b^	0.74 ± 0.03 ^d^	0.84 ± 0.02 ^bc^	0.81 ± 0.03 ^c^	0.84 ± 0.02 ^bc^	0.81 ± 0.01 ^c^	0.96 ± 0.01 ^a^
**Phenylalanine**	0.58 ± 0.02 ^b^	0.50 ± 0.02 ^c^	0.58 ± 0.03 ^b^	0.56 ± 0.02 ^b^	0.58 ± 0.01 ^b^	0.57 ± 0.00 ^b^	0.67 ± 0.01 ^a^
**Threonine**	0.37 ± 0.02 ^ab^	0.32 ± 0.02 ^c^	0.36 ± 0.02 ^ab^	0.36 ± 0.01 ^ab^	0.35 ± 0.02 ^bc^	0.34 ± 0.01 ^bc^	0.39 ± 0.02 ^a^
**Methionine**	0.06 ± 0.01 ^ab^	0.06 ± 0.00 ^ab^	0.05 ± 0.01 ^ab^	0.05 ± 0.00 ^ab^	0.06 ± 0.01 ^b^	0.06 ± 0.00 ^ab^	0.07 ± 0.00 ^a^
**Lysine**	0.64 ± 0.02 ^b^	0.58 ± 0.03 ^d^	0.63 ± 0.02 ^bc^	0.61 ± 0.02 ^c^	0.63 ± 0.01 ^bc^	0.61 ± 0.01 ^c^	0.69 ± 0.01 ^a^
**Histidine**	0.22 ± 0.01 ^a^	0.20 ± 0.01 ^ab^	0.20 ± 0.02 ^ab^	0.20 ± 0.01 ^ab^	0.19 ± 0.01 ^b^	0.19 ± 0.01 ^b^	0.21 ± 0.02 ^a^
**Isoleucine**	0.40 ± 0.01 ^b^	0.34 ±0.01 ^d^	0.38 ± 0.01 ^bc^	0.37 ± 0.01 ^c^	0.39 ± 0.00 ^b^	0.37 ± 0.01 ^c^	0.44 ± 0.01 ^a^
**Non-Essential Amino Acids (NEA)**							
**Aspartic acid**	1.04 ± 0.02 ^b^	0.94 ± 0.03 ^c^	1.04 ± 0.04 ^b^	1.04 ± 0.04 ^b^	1.00 ± 0.02 ^b^	1.02 ± 0.01 ^b^	1.16 ± 0.02 ^a^
**Valine**	0.57 ± 0.04 ^b^	0.49 ± 0.03 ^c^	0.55 ± 0.03 ^b^	0.54 ± 0.02 ^b^	0.56 ± 0.02 ^b^	0.54 ± 0.01 ^b^	0.62 ± 0.02 ^a^
**Alanine**	0.48 ± 0.01 ^b^	0.43 ± 0.01 ^c^	0.48 ± 0.02 ^b^	0.48 ± 0.02 ^b^	0.48 ± 0.01 ^b^	0.47 ± 0.00 ^b^	0.57 ± 0.01 ^a^
**Glycine**	0.64 ± 0.01 ^b^	0.58 ± 0.01 ^d^	0.65 ± 0.02 ^b^	0.63 ± 0.03 ^bc^	0.61 ± 0.01 ^cd^	0.61 ± 0.02 ^cd^	0.70 ± 0.02 ^a^
**Proline**	0.52 ± 0.01 ^b^	0.46 ± 0.01 ^d^	0.52 ± 0.02 ^b^	0.51 ± 0.02 ^b^	0.51 ± 0.01 ^b^	0.48 ± 0.01 ^c^	0.59 ± 0.01 ^a^
**Glutamic acid**	2.13 ± 0.08 ^b^	1.84 ± 0.06 ^d^	2.13 ± 0.11 ^b^	1.94 ± 0.07 ^cd^	2.05 ± 0.02 ^bc^	2.00 ± 0.01 ^c^	2.40 ± 0.03 ^a^
**Tyrosine**	0.24 ± 0.01 ^b^	0.22 ± 0.01 ^c^	0.26 ± 0.02 ^a^	0.26 ± 0.01 ^a^	0.24 ± 0.01 ^b^	0.24 ± 0.02 ^b^	0.27 ± 0.01 ^a^
**Serine**	0.50 ± 0.01 ^b^	0.45 ± 0.01 ^c^	0.52 ± 0.02 ^b^	0.52 ± 0.03 ^b^	0.49 ± 0.01 ^b^	0.49 ± 0.02 ^b^	0.55 ± 0.02 ^a^
**ΣEAA**	3.75 ± 0.10	3.28 ± 0.06	3.68 ± 0.11	3.59 ± 0.14	3.65 ± 0.03	3.53 ± 0.05	4.08 ± 0.06
**ΣNEA**	6.13 ± 0.16	5.42 ± 0.15	6.16 ± 0.23	5.90 ± 0.20	5.93 ± 0.06	5.85 ± 0.04	6.85 ± 0.08
**ΣEAA/ΣNEA**	0.61 ± 0.01	0.60 ± 0.01	0.60 ± 0.01	0.61 ± 0.01	0.62 ± 0.01	0.60 ± 0.00	0.59 ± 0.01
TAA	9.88 ± 0.25	8.70 ± 0.20	8.83 ± 0.33	9.49 ± 0.33	9.58 ± 0.07	9.38 ± 0.09	10.59 ± 0.13

EAA, essential amino acids; NEA, nonessential amino acids; TAA, total amino acids; Means with different letters within the same row are significantly different (*p* < 0.05).

### 2.7. Volatile Flavor Compounds Identified from Chinese Robusta Coffee

**Figure 3:**
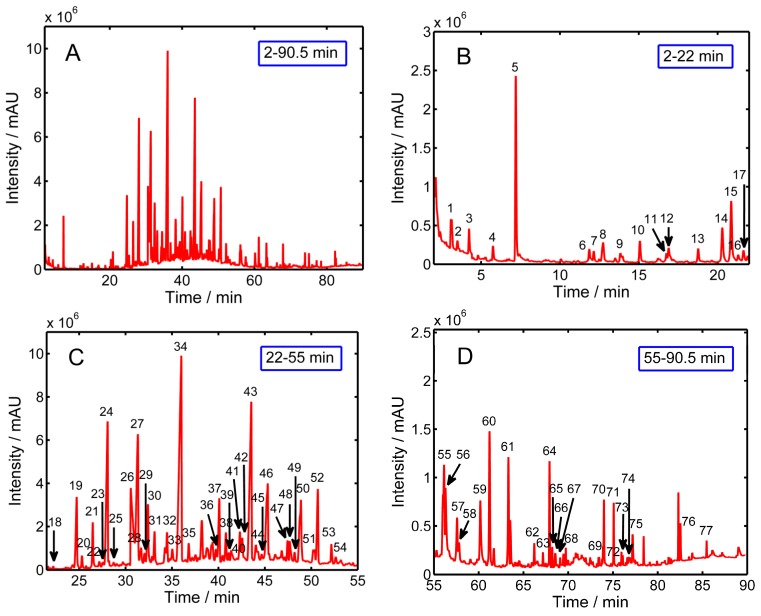
Characteristic GC-MS chromatogram of the volatile compounds from coffee samples, ((**A**): 2–90.5 min; (**B**): 2–22 min; (**C**): 22–55 min; (**D**): 55–90.5 min).

Table 4 lists the volatile compounds detected in green coffee beans by the HS-SPME/GC-MS technique in order of their retention time. In all, 79 compounds were identified and grouped into 11 chemical groups, including seven alcohols, five acids, 30 hydrocarbons, 14 aldehydes, nine esters, one furan, three pyrazines, one pyridine, four ketones, two phenols and four others. Volatile composition of the seven cultivars by chemical class is presented in Table 5, and a representative GC-MS chromatogram of the volatiles in green coffee beans is shown in Figure 3A–D. Many of these compounds have been previously identified in Brazilian coffees as well as other coffee samples [28,29]. All the identified volatile compounds constituted an average of 82% of the total peak area, and thus represented the majority of the compound profiles. Also, there was no specific volatile compound that was found in a single cultivar. In general, cultivar flavor differences are due to quantitative differences, not qualitative differences. Hydrocarbons represented the largest volatile group in the coffee samples examined in this study, and the total hydrocarbon content accounted for 54.9% (“X26”) to 63.20% (“RY1”) of the total volatiles. Four hydrocarbons—tetradecane, 7-methylpentadecane, pentadecane and hexadecane—were present in higher amounts when compared to other hydrocarbons. Next were acids and aldehydes, which ranged from 4.84% (“RY2”) to 7.22% (“X1”) and 5.03% (“RY1”) to 6.05% (“XCM”) of total volatiles, whereas esters and alcohols were in intermediate amounts, with values from 2.73% (“X28”) to 3.44% (“RY1”) and 2.08% (“RY1”) to 4.83% (“XCM”). The remaining chemical classes such as furans, pyrazines, pyridines, ketones and phenols were only found in small or trace amounts. It is noticed that volatile differences were not significant for some cultivars. Cheong *et al.* [45] investigated and compared the volatile constituents of four Asian coffee varieties and 62 volatile compounds were identified. Knowledge of the volatile composition would facilitate a better understanding of Asian coffee quality. In our study, only applying ANOVA to the volatile composition data to examine the differences provided incomplete results, thus, in order to verify whether the volatile profiles could be applied to discriminate green coffee beans from different cultivars and to investigate the volatiles that contributed the most to the separation between samples, multivariate statistical analyses, PCA and HCA were applied to the complete data set.

**Table 4:** Volatile compounds identified in different coffee (*Coffea Robusta*) cultivars detected by HS-SPME/GC-MS methodology.

No.	R_t_ (min)	Compounds	RI	Identification	Formula	Mol. Wt
1	3.080	3-Methylbutanal	912	MS, RI	C_5_H_10_O	86
2	3.483	Ethanol	944	MS, RI	C_2_H_6_O	46
3	4.232	Pentanal	978	MS, RI	C_5_H_10_O	86
4	5.744	Toluene	1008	MS, RI	C_7_H_8_	92
5	7.195	Hexanal	1053	MS, RI	C_6_H_12_O	94
6	11.853	Pyridine	1159	MS, RI	C_5_H_5_N	79
7	12.141	Heptanal	1165	MS, RI	C_7_H_14_O	114
8	12.743	d-Limonene	1176	MS, RI	C_10_H_16_	94
9	13.833	2-Hexenal	1197	MS, RI	C_6_H_10_O	98
10	15.072	2-Pentylfuran	1216	MS, RI	C_9_H_14_O	138
11	16.752	1-Pentanol	1240	MS, RI	C_5_H_12_O	88
12	16.892	Methylpyrazine	1243	MS, RI	C_5_H_6_N_2_	94
13	18.769	Octanal	1270	MS, RI	C_8_H_16_O	128
14	20.298	Tridecane	1294	MS, RI	C_13_H_28_	184
15	20.867	(*Z*)-2-Heptenal	1301	MS, RI	C_7_H_12_O	112
16	21.307	2,6-Dimethylpyrazine	1307	MS, RI	C_6_H_8_N_2_	108
17	21.644	Ethylpyrazine	1312	MS, RI	C_6_H_8_N_2_	108	
18	22.213	6-Methyl-5-hepten-2-one	1319	MS, RI	C_8_H_14_O	126	
19	24.737	2,6,10,14-Tetramethylhexadecane	1353	MS, RI	C_20_H_42_	282	
20	25.310	3,5-Dimethyldodecane	1360	MS, RI	C_14_H_30_	198	
21	26.465	Nonanal	1376	MS, RI	C_9_H_18_O	142	
22	27.177	4,8-Dimethyltridecane	1385	MS, RI	C_15_H_32_	212	
23	27.565	(*E*)-3-Tetradecene	1390	MS, RI	C_14_H_28_	196	
24	28.071	Tetradecane	1397	MS, RI	C_14_H_30_	198	
25	28.713	2-Dodecenal	1405	MS, RI	C_12_H_22_O	182	
26	30.565	Acetic acid	1429	MS, RI	C_2_H_4_O_2_	60	
27	31.336	7-Methylpentadecane	1439	MS, RI	C_16_H_34_	226	
28	31.679	1-Octen-3-ol	1444	MS, RI	C_8_H_16_O	128	
29	32.052	4-Methyltetradecane	1449	MS, RI	C_15_H_32_	212	
30	32.421	10-Methyleicosane	1453	MS, RI	C_21_H_44_	296	
31	33.129	3-Methyltetradecane	1463	MS, RI	C_15_H_32_	212	
32	34.471	1,1′-(1,3-Propanediyl)bis-cyclohexane	1480	MS, RI	C_15_H_28_	208	
33	35.032	Benzaldehyde	1487	MS, RI	C_7_H_6_O	106	
34	36.022	Pentadecane	1500	MS, RI	C_15_H_32_	212	
35	36.805	2-Methyl-(*Z*)-4-tetradecene	1510	MS, RI	C_15_H_30_	210	
36	39.743	4-Methyldodecane	1549	MS, RI	C_13_H_28_	184	
37	40.108	2-Methylpentadecane	1554	MS, RI	C_16_H_34_	226	
38	40.821	Benzeneacetic acid-2-tetradecyl ester	1563	MS, RI	C_22_H_36_O_2_	332	
39	41.121	2-Tridecanol	1567	MS, RI	C_13_H_28_O	200	
40	41.383	(*Z*)-3-Hexadecene	1570	MS,RI	C_16_H_32_	224	
41	42.313	Butyrolactone	1583	MS, RI	C_4_H_6_O_2_	86	
42	42.580	*n*-Nonylcyclohexane	1586	MS, RI	C_15_H_30_	210	
43	43.565	Hexadecane	1599	MS, RI	C_16_H_34_	226	
44	44.033	Benzeneacetaldehyde	1605	MS, RI	C_8_H_8_O	120	
45	44.672	1-Heptacosanol	1614	MS, RI	C_27_H_56_O	396	
46	45.333	2,6,10-Trimethylpentadecane	1623	MS, RI	C_18_H_38_	254	
47	47.460	Methoxyacetic acid-2-tetradecyl ester	1652	MS, RI	C_17_H_30_O_3_	286	
48	47.687	3-Methylbutanoic acid	1655	MS, RI	C_5_H_10_O_2_	102	
49	48.213	3-Methylhexadecane	1662	MS, RI	C_17_H_36_	240	
50	48.907	2,6,10,14-Tetramethylpentadecane	1672	MS, RI	C_19_H_40_	268	
51	50.221	Naphthalene	1690	MS, RI	C_10_H_8_	128	
52	50.726	Heptadecane	1697	MS, RI	C_17_H_36_	240	
53	52.640	2-Methyl-(*E*)-7-octadecene	1724	MS, RI	C_19_H_38_	266	
54	53.183	Methyl salicylate	1731	MS, RI	C_8_H_8_O_3_	152	
55	56.140	2,6,10,14-Tetramethylhexadecane	1773	MS, RI	C_20_H_42_	282	
56	56.296	3-Methyl-2-butenoic acid	1776	MS, RI	C_5_H_8_O_2_	100	
57	57.592	Octadecane	1794	MS, RI	C_18_H_38_	254	
58	57.812	2-Cyclohexyldecane	1797	MS, RI	C_16_H_32_	224	
59	60.186	Hexanoic acid	1833	MS, RI	C_6_H_12_O_2_	116	
60	61.697	2,2,4-Trimethyl-3-carboxy isopropylpentanoic acid, isobutyl ester	1857	MS, RI	C_6_H_30_O_4_	286	
61	63.323	Phenylethyl alcohol	1882	MS, RI	C_8_H_10_O	122	
62	66.212	1-(1*H*-pyrrol-2-yl)-ethanone	1940	MS, RI	C_6_H_7_NO	109	
63	67.180	1-Tetradecanol	1962	MS, RI	C_14_H_30_O	214	
64	67.914	Phenol	1978	MS, RI	C_6_H_6_O	94	
65	68.217	Dihydro-5-pentyl-2(3*H*)-furanone	1985	MS, RI	C_9_H_16_O_2_	156	
66	68.556	2-Pyrrolidinone	1993	MS, RI	C_4_H_7_NO	85	
67	69.107	Octadecanal	2008	MS, RI	C_18_H_36_O	268	
68	69.481	Eicosane	2019	MS, RI	C_17_H_36_	240	
69	73.400	Caprolactam	2142	MS, RI	C_6_H_11_NO	113	
70	73.992	2-Methoxy-4-vinyphenol	2163	MS, RI	C_9_H_10_O_2_	150	
71	75.083	Hexadecanoic acid methyl ester	2202	MS, RI	C_17_H_34_O_2_	270	
72	75.954	2-Methyl-2-cyclopenten-1-one	2237	MS, RI	C_6_H_8_O	96	
73	76.094	Hexadecanoic acid ethyl ester	2243	MS, RI	C_18_H_36_O_2_	284	
74	76.746	*n*-Decanoic acid	2268	MS, RI	C_10_H_20_O_2_	172	
75	77.221	5,6,7,7a-Tetrahydro-4,4,7a-trimethyl-2(4*H*)-benzofuranone	2287	MS, RI	C_11_H_16_O_2_	180	
76	82.546	3-Hydroxy-4-methoxybenzaldehyde	2527	MS, RI	C_8_H_8_O_3_	152	
77	85.466	1,2-Benzenedicarboxylic acid-butyl-2-methylpropyl ester	2669	MS, RI	C_16_H_22_O_4_	278	

**Table 5:** Comparison of volatile compounds according to chemical classes semi-quantified in seven cultivars of Robusta coffee.

Code	Relative Peak Areas of Samples (%)						
	X1	RY1	RY2	X24-2	X26	X28	XCM
**Alcohols**	2.72 ± 0.22 ^bc^	2.08 ± 0.24 ^c^	2.18 ± 0.11 ^c^	2.41 ± 0.12 ^bc^	2.24 ± 0.15 ^bc^	2.95 ± 0.29 ^b^	4.83 ± 0.65 ^a^
**Acids**	7.22 ± 0.69 ^ab^	6.79 ± 0.12 ^abc^	4.84 ± 1.91 ^c^	4.89 ± 0.40 ^c^	8.85 ± 0.13 ^a^	6.50 ± 0.68 ^bc^	6.87 ± 0.90 ^ab^^c^
**Hydrocarbons**	61.07 ± 2.02 ^a^	63.20 ± 1.77 ^a^	61.87 ± 1.93 ^a^	62.70 ± 4.50 ^a^	54.90 ± 0.39 ^b^	60.33 ± 1.08 ^a^	44.60 ± 2.08 ^c^
**Aldehydes**	5.55 ± 0.57 ^ab^	5.03 ± 0.20 ^b^	5.45 ± 0.10 ^b^	5.27 ± 0.08 ^b^	5.17 ± 0.10 ^b^	5.28 ± 0.05 ^b^	6.05 ± 0.27 ^a^
**Esters**	3.13 ± 0.04 ^ab^	3.44 ± 0.26 ^a^	3.01 ± 0.18 ^ab^	3.12 ± 0.20 ^ab^	3.26 ± 0.30 ^a^	2.73 ± 0.19 ^b^	2.94 ± 0.25 ^ab^
**Furan**	0.12 ± 0.02 ^b^	0.12 ± 0.01 ^b^	0.11 ± 0.01 ^b^	0.11 ± 0.01 ^b^	0.17 ± 0.02 ^b^	0.15 ± 0.03 ^b^	0.27 ± 0.02 ^a^
**Pyrazines**	0.23 ± 0.04 ^a^	0.72 ± 0.09 ^b^	0.14 ± 0.04 ^a^	0.16 ± 0.02 ^a^	0.30 ± 0.02 ^a^	0.49 ± 0.11 ^c^	2.96 ± 0.13 ^a^
**Pyridine**	0.10 ± 0.02 ^c^	0.33 ± 0.02 ^b^	0.10 ± 0.03 ^c^	0.08 ± 0.01 ^c^	0.10 ± 0.01 ^c^	0.17 ± 0.04 ^c^	1.48 ± 0.10 ^a^
**Ketones**	1.31 ± 0.03 ^c^	1.01 ± 0.08 ^c^	1.17 ± 0.04 ^c^	1.08 ± 0.39 ^c^	3.55 ± 0.19 ^a^	1.30 ± 0.10 ^c^	2.92 ± 0.16 ^b^
**Phenols**	0.63 ± 0.06 ^bc^	0.80 ± 0.08 ^b^	0.76 ± 0.08 ^bc^	0.69 ± 0.07 ^bc^	0.50 ± 0.24 ^c^	0.78 ± 0.09 ^b^	1.06 ± 0.05 ^a^
**Others**	0.41 ± 0.15 ^a^	0.25 ± 0.06 ^ab^	0.23 ± 0.16 ^ab^	0.10 ± 0.05 ^b^	0.27 ± 0.17 ^ab^	0.29 ± 0.06 ^ab^	0.42 ± 0.03 ^a^

Means with different letters within the same row are significantly different (*p* < 0.05).

### 2.8. Principal Component Analysis 

**Figure 4:**
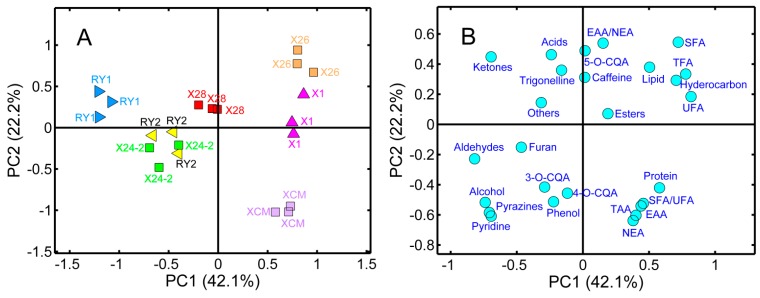
Projection of the samples on the plane defined by the first and second principal components (**A**) and the corresponding loading plot (**B**) using the combined data matrix (21 samples × 26 variables) as the analytical data set.

PCA is an unsupervised pattern recognition method, which minimizes the possibility of losses of information in a dataset by reducing the data set dimensionality, and can preserve the original variability by linear combination of the variables. In general, the first few principal components (PCs) could explain most of the variability in a classification. Coefficients by which the original variables are multiplied to obtain the PC are called loadings, whose numerical values show how similar each variable is compared to that component, the distribution of samples for different cultivars can be visualized in PCA two or three-dimensional (2D or 3D) plots defined by PCs [46,47]. PCA was performed on the complete data matrix (21 samples × 26 variables), where the variables comprised the fatty acids (SFA, UFA, SFA/UFA and TFA), the amino acids (EAA, NEA, EAA/NEA and TAA), the lipid and protein contents, the bioactive components (3-*O*-CQA, 5-*O*-CQA, 4-*O*-CQA, trigonelline, and caffeine), the 11 volatile chemical groups (alcohols, acids, hydrocarbons, aldehydes, esters, furan, pyrazines, pyridine, ketones, phenols and others). The first three PCs were sufficient to describe 76.5% of the variation, with values of 42.1%, 22.2%, and 12.1%, respectively. Figure 4 presents a scatter plot (A: score; B: loading) of the first two PCs, it can be observed that different cultivars can be separated in the 2-D biplot except for the sample overlap between the “RY2” and “X24-2” cultivars. When all samples were projected on the PC1 axis, “X26”, “X1”, and “XCM” had positive scores on PC1, whereas “X28” was located almost in the origin of coordinates, other samples “RY1”, “X24-2”, and “RY2” had negative scores on PC1, thus, “X26”, “X1”, and “XCM” were well separated from others, and the same ocurred for “RY1”. When all samples were projected on the PC2 axis, “X26”, “RY1”, and “X28” had positive scores on this direction, two samples for “X1” and “RY2” were around a value of zero, respectively, and “X24-2” and “XCM” had negative scores on PC2, therefore, “X26” (highest positive score) and “XCM” (highest negative score) could be discriminated from other samples which exhibited relatively intermediate scores on the PC2 direction. By using a combination of PC1 and PC2, most samples were well separated from each other, except for “RY2” and “X24-2” with minor overlap, this phenomenon can be attributed to their similar chemical profiles when all components considered were taken into account in this study. Figure 4B shows the PCA loading plot, where SFA, UFA, and SFA/UFA, as well as the hydrocarbons, had positive loadings on PC1, “X26” and “X1” had high scores on PC1, indicating high quantities of these fatty acids and hydrocarbons. Inversely, ketones and aldehydes with negative loadings on PC1, indicating they were correlated with the “RY1” samples. On the other hand “XCM” samples were located in the fourth quadrant, characterized by the most EAAs, NEAs, and TAAs, as well as SFA/UFA and protein contents. The amounts of furan with lower negative values on PC1 and PC2 were substantially higher in “RY2” and “X24-2”. PCA was also applied to discriminate coffee samples from different geographical origins (Thailand, Indonesia, and China) using volatile compositions as the input variables, and a PC1-PC2 bioplot suggested a significant difference in the aroma profiles between each other [45]. Volatiles extraction and concentration combined with PCA could differentiate healthy and defective Brazilian coffee beans [48].

### 2.9. Hierarchical Cluster Analysis

**Figure 5:**
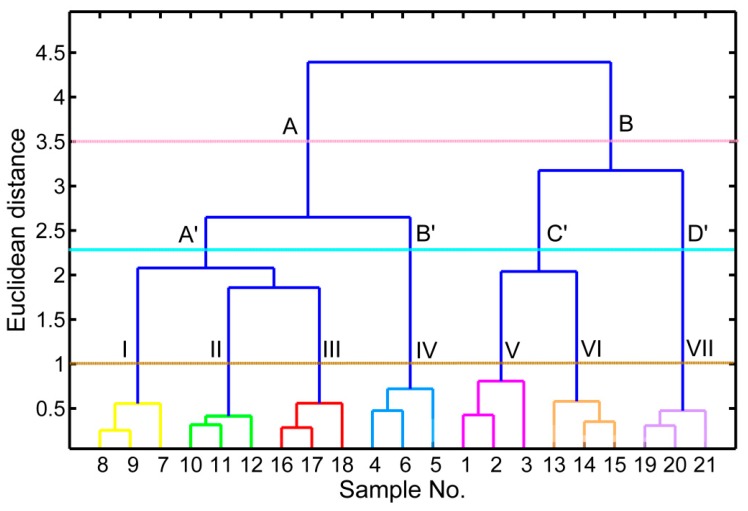
Hierarchical dendrogram constructed from the combined data matrix (21 samples × 26 variables) as the input variables, illustrating the distances between seven coffee cultivars.

In order to better visualize the samples’ structure and variety similarity, HCA was used as a complementary multivariate analysis approach to evaluate the relationships between the samples of different origins. HCA using Ward’s linkage based on the composition of complete data set was performed. The HCA is also an unsupervised pattern recognition technique that allows the classification of variables into groups based on the Euclidean distances between samples [49]. A dendrogram of the 21 coffee samples is presented in Figure 5. All cultivars fell into a single subgroup. The replicates of each cultivar were clustered into one group, indicating that the biological variation among samples harvested at different locations was less than the cultivar-to-cultivar variance. Two major clusters, *viz*. clusters A and B, can be found in Figure 5. Cluster A consists of 12 samples, while cluster B was composed of nine samples. Cluster A formed two subclusters, *viz*., A′, B′, and C′, D′. It can be observed that cluster A′ was split into three primary subclades (I: “RY2”; II: “X24-2”; III: “X28”), cluster B′ contained IV (“RY1”); Cluster C′ sorts coffee cultivars into two clades (V: “X1”; VI: “X26”), whereas cluster D′ contained one group (VII: “XCM”). Relationships between the analyzed cultivars were similar to those obtained by PCA, indicating that chemometric analysis combined with chemical composition could be used as an effective tool to explore the phylogenetic relationships among these species. HCA analysis in combination with ESI (–)-MS results indicated that the Arabica and Robusta coffee samples were separated into two distinct clusters [50]. Those results showed that HCA was an effective method for the quality control of coffee samples.

## 3. Experimental Section 

### 3.1. Chemicals and Reagents

Trigonelline, caffeine, 3-caffeoylquinic acid (3-CQA), 4-caffeoylquinic acid (4-CQA), 5-caffeoylquinic acid (5-CQA), standards of fatty acid methyl ester (FAME) mixture, amino acid standards, alkanes (C6-C40) were acquired from Sigma-Aldrich (St. Louis, MO, USA). Methanol for ultra-phase liquid chromatography (UPLC grade) and glacial acetic acid were obtained from Merck (Darmstadt, Germany). UPLC grade water was prepared by Millipore purification and filtration system (Master-s-plus UVF, Shanghai, China), and additionally filtrated through a 0.22 μm membrane filter (ANPEL Scientific Instrument, Shanghai, China) before use. All others chemicals were of analytical or UPLC grade.

### 3.2. Coffee Samples and Sample Preparation

Twenty-one green coffee samples belonging to seven different cultivars were compared and evaluated, each cultivar contained three samples. Seven cultivars of *C*. *canephora* (Robusta coffee) were named as Robusta Xinglong 1 (“X1”), No. 1–3, Robusta Reyan 1 (“RY1”), No. 4–6, Robusta Reyan 2 (“RY2”) No. 7–9, Robusta Xinglong 24-2 (“X24-2”), No. 10–12, Robusta Xinglong 26 (“X26”), No. 13–15, Robusta Xinglong 28 (“X28”), No. 16–18, Robusta Chenmai (“XCM’), No. 19–21, respectively, all of above cultivars are mainly commercially cultivated in Hainan Province, China. All of the plants are maintained in the Coffee Germplasm Bank and are cultivated in the experimental center of the Spice and Beverage Research Institute, Chinese Academy of Tropical Agricultural Sciences (CATAS), Wanning, China. Coffee fruits were harvested in the cherry maturation stage during the 2014/2015. After harvesting, fresh coffee fruits were mechanically hulled and removed the mucilage, additionally coffee endosperms with the parchment were subjected to hot air drying in an oven (Model 101-2-BS, Shanghai Yuejin Medical Instrument Co. Ltd., Shanghai, China) at 50 °C until the moisture content were about 10%–11%/100 g DW. And the parchment were mechanically removed, endosperms were ground into powder using an grinder, and passed through an 40 mesh sieve, vacuum packed in foil bag, and stored in the dark at 4 °C for further analysis.

### 3.3. Physicochemical Analyses.

#### 3.3.1. Color Measurements

The color of each sample was measured by ground coffee bean light reflectance using a model SP62 X-rite spectrophotometer (X-Rite, Incorporated, Grand Rapids, MI, USA), the CIELAB system was used and values of L* (lightness), a* (red-green component), and b* (yellow-blue component) were acquired from the equipment. Before analysis, the instrument was calibrated on a white standard tile, hue angle (h°) was calculated using values of L*, a*, and b*, and can be expressed as h° = tan^−1^ (b*/a*). The colorimetric difference between two samples was calculated using the equation:

ΔE* = [(L* − L_o_*)^2^ + (a* − a_o_*)^2^ + (b* − b_o_*)^2^]^1/2^ (1)

The illuminant D65/10° was used and specular component excluded, measurement of color was performed on five randomly chosen spots on each sample.

#### 3.3.2. Preparation of Sample Solutions for Chlorogenic Acids, Trigonelline, and Caffeine Determination 

For chlorogenic acids analysis, powdered coffee samples (0.4 g) were accurately weighed into a 50 mL conical flask, then 70% ethanol/water (20 mL) was added, the mixture was refluxed three times at 70 °C for 2.5 h, and the combined solution was filtered and evaporated under vacuum, the filtrate were diluted into a 50 mL volumetric flask. The mixture was filtered through 0.22 μm PET syringe membranes before UPLC analysis. As far as trigonelline and caffeine determination are concerned, sample (0.2 g) was first put into a 100 mL conical flask, then the sample was extracted three times with deionized water (30 mL) for 60 min at 95 °C, and filtered through #202 filter paper (Wohua Co., Hangzhou, China), filtrates were combined into a 100 mL volumetric flask and diluted to volume using deionized water [31]. Similarly, water extracts were filtered through 0.22 μm regenerated cellulose membrane prior to UPLC analysis.

#### 3.3.3. UPLC-DAD Analysis

Coffee extracts analysis was performed with an Agilent 1290 series UPLC system (Agilent Technologies, Inc., Santa Clara, CA, USA), equipped with a G4204A quaternary pump, a G4226A autosampler, a G1316C column oven, and a G4212A DAD detector. The chromatography was carried out on an Alltech Alltima C-18 column (2.1 × 100 mm, 1.7 μm). The mobile phases consisted of A (0.1% acetic acid in deionized) and C (methanol). A linear gradient elution procedure was used: 0–30 min, 5%–100% C; 30–32 min, 100%–100% C. The flow rate was set as 0.2 mL/min and injection volume was 1.5 μL, and the post run equilibration time was 7 min. Quantification was performed by the external standard method, calibration curve was established by linear regression based on corresponding standard solution. Quantification of compounds was conducted using a DAD detector by integrating peak areas at 325 nm for CGAs, 268 nm for trigonelline, and 275 nm for caffeine. The data analysis was performed using the Agilent ChemStation software.

#### 3.3.4. Determination of Total Protein Content

The total protein content determination was conducted using the Dumas system (NDA 701, Velp Scientifica, Milan, Italy), whereby crude protein content was calculated as nitrogen content multiplied by a factor of 6.25. Samples were first freeze-dried and then, samples (100 mg) were accurately weighed and submitted to routine analysis. The operating conditions of the Dumas system were as follows: flow rates for O_2_ and He were 400 and 195 mL/min, respectively; pressures for He, O_2_, and N_2_ were 2.0, 2.5, and 3.0 bar, respectively; combustion reactor: 1030 °C; reduction reactor: 650 °C. The total protein content was expressed as g/100 g dry weight of sample (g/100 g DW). All measurements were performed in triplicate.

#### 3.3.5. Determination of Total Lipid Content

Lipid extraction was carried out using the procedure described by Xiao *et al.* [51] with minor modifications. Briefly, accurately weighed freeze-dried samples (3.0 g) were packed with cotton and added into the chamber of pre-dried Soxhlet fat extraction system. Approximately 120 mL of petroleum ether (fraction 30–60 °C) was added to the sample and mixed, then the extraction was carried out at 50 °C for about 16–18 h until the petroleum ether in the extraction tube was colorless. After extraction, the solvent in the extract was removed with rotary evaporation at 40 °C, and the extract was dried in an oven at 105 °C, then cooled in a desiccator before gravimetric fat determination, dry weight of the resulting extract was applied to calculate the total lipid content of coffee sample. The total lipid content was expressed as g/100 g dry weight of sample (g/100 g DW). All measurements were made in triplicate.

#### 3.3.6. Fatty Acid Analysis 

Fatty acid methyl esters were obtained by transmethylation with 2 M KOH in anhydrous methanol according to the ISO 12966-2 standard [52]. Fatty acids were analysed by using an Agilent 7890A gas chromatograph equipped with flame ionization detector (FID) with split/splitless injector, the split ratio was 1:50. Fatty acid separation was performed on a HP-88 column (Agilent Technologies). Helium was used as carrier gas at a flow rate of 1.0 mL/min, and the injector port and detector temperatures were set at 250 °C and 260 °C, respectively. The oven temperature program was as follows, 100 °C was kept for 2 min, ramped to 170 °C at 20 °C/min, and rised to 200 °C at 2 °C/min and held for 10 min, then raised to 230 °C and held for 5 min. The identifications of the peaks were performed by comparing the retention times of the sample with a certified FAME mix and by comparison with literature reports with regard to coffee oil. Fatty acids were quantitative analyzed and presented as weight percentage (g/100 g DW sample). All measurements were performed in triplicate.

#### 3.3.7. Amino Acid Analysis 

Briefly, ground samples (100 mg) were hydrolysed with 6 M HCl (10 mL) in sealed, evacuated glass tubes at 110 °C for 22 h. After hydrolysis, the mixtures were filtered through #202 filter paper and a portion of the filtrate (0.5 mL) was transferred into a 10 mL round-bottom flask and evaporated by rotary evaporation. The hydrolysate was resuspended in diluent (3.0 mL) and filtered through a 0.22 μm microfiltration membrane, free amino acids in filtrate were determined using a reversed-phase high performance liquid chromatography SYKAM Amino Acid Analyzer S-433D (Sykam GmbH; Kleinostheim, Germany) with a PEEK column (4.6 × 150 mm, 7 μm, 10% crosslink) to determine the free amino acid. The content of free amino acids was calculated by calibrating with standard amino acids and expressed as g/100 g sample. All measurements were performed in triplicate.

#### 3.3.8. Extraction of Volatile Compounds from Ground Coffee by HS-SPME

The volatile composition was analyzed by headspace-solid phase microextraction (HS-SPME) coupled to gas chromatography with mass spectrometry (MS) according to the method of Bertrand *et al.* [28] with minor revisions. A carboxen/polymethylsiloxane (CAR/PDMS, 75 μm) SPME fibre (Supelco Co., Bellefonte, PA, USA) was used for headspace analyses of coffee samples volatile since its affinities in analysis for all classes of aroma components of coffee which has been widely reported in previous studies [53,54]. Two grams of samples were placed into a 20-mL glass vials sealed with PTFE-silicone septa supplied by Supelco, the sample vial was allowed to equilibrate at 60 °C for 20 min. The SPME fibre was in contact with the headspace to extract the volatiles for 40 min with continuous heating. After extraction, the fibre was transferred to the injector port for desorption at 250 °C for 4 min.

#### 3.3.9. GC-MS Analysis and Volatile Compounds Identification

GC-MS analysis was performed on an Agilent 7890A gas chromatography and an Agilent 5975C Mass Selective Detector. GC separation was carried out on a polar polyethylene glycol phase (DB-WAX, 30 m × 0.25 mm, d_f_ = 0.25 μm, Agilent J&W Columns, Santa Clara, CA, USA), helium was used as carrier gas at a constant flow of 1.0 mL/min. The oven temperature program was as follows: isothermal hold at 40 °C for 2 min, then rise to 130 °C (1.5 °C/min), constant rise to 220 °C (4 °C/min) held for a further 5 min. The electron impact ionization, quadrupole, and transfer line temperature were set to 250, 150, and 250 °C, respectively. Mass spectra and total ion chromatograms were recorded in full-scan mode from *m/z* 50 to 350 at a rate of 3.06 scan/s. Individual compounds identification was based on a comparison of their mass spectra and retention times with those of authentic standards, or by comparison of retention indices (RI) with the NIST08 library, the RI.s were calculated from the retention times of C7-C30 *n*-alkanes. Tentative identification was based on matching mass spectra of unknowns with those in NIST08 library. Relative quantification of volatile compounds was carried out by integrating the peak areas of each extracted for each analyte and expressed as their respective percentage content, the obtained relative concentration was subject to difference analysis in volatile profiles among different coffee cultivars. The experiment was made in triplicate.

### 3.4. Statistical Analysis

All statistical analyses were performed using SPSS version 20.0 software (SPSS Inc., Chicago, IL, USA) and MATLAB R2010a software (The MathWorks Inc., Natick, MA, USA). Significant differences among physicochemical characteristics and volatiles of different cultivars, respectively, were verified by one-way analysis of variance (ANOVA), Duncan’s multiple comparison were used to compare means statistically significant difference between samples at a 95% confidence level. Principal component analysis (PCA) and hierarchical cluster analysis (HCA) were applied to visualize the differences in the analyzed components between samples in two-dimensional space and identify variables which were responsible for sample separation. In order to avoid the effect of different scales of the variables, all data set were auto-scaled prior to the analysis. The results were presented as the mean ± SD (standard deviation) of triple measurements. 

## 4. Conclusions

In conclusion, the present study demonstrated that seven Robusta coffee cultivars grown in China presented different physicochemical characteristics that could be used to distinguish coffee cultivars in regard to their flavor quality and potential uses. The results showed that significant differences exist in fatty acids, amino acids and volatile compounds composition as well as bioactive components (chlorogenic acids, trigonelline and caffeine) among the different coffee cultivars, and linoleic acid, palmitic acid, oleic acid, and arachic acid were the major fatty acids. Leucine, lysine, and arginine were the predominant EAAs in the coffee samples. A total of seventy-seven volatiles were identified and hydrocarbons, acids, aldehydes, and alcohols accounted for more than 90% of the total volatile compounds. PCA and HCA chemometric methods of analysis could classify the samples into seven groups and produce acceptable performance.
